# A complex structural variant near *SOX3* causes X-linked split-hand/foot malformation

**DOI:** 10.1016/j.xhgg.2023.100200

**Published:** 2023-04-25

**Authors:** Elke de Boer, Carlo Marcelis, Kornelia Neveling, Ellen van Beusekom, Alexander Hoischen, Willemijn M. Klein, Nicole de Leeuw, Tuomo Mantere, Uirá S. Melo, Jeroen van Reeuwijk, Dominique Smeets, Malte Spielmann, Tjitske Kleefstra, Hans van Bokhoven, Lisenka E.L.M. Vissers

**Affiliations:** 1 Department of Human Genetics, Radboudumc University Medical Center, Nijmegen, the Netherlands; 2 Donders Institute for Brain, Cognition and Behaviour, Radboud University, Nijmegen, the Netherlands; 3 Department of Internal Medicine and Radboud Center for Infectious Diseases, Radboud University Medical Center, Nijmegen, the Netherlands; 4 Radboud Institute for Molecular Life Sciences, Radboud University Medical Center, Nijmegen, the Netherlands; 5 Department of Medical Imaging, Radiology, Radboud University Medical Center, Nijmegen, the Netherlands; 6 Laboratory of Cancer Genetics and Tumor Biology, Cancer and Translational Medicine Research Unit and Biocenter Oulu, University of Oulu, Oulu, Finland; 7 Max Planck Institute for Molecular Genetics, RG Development & Disease, Berlin, Germany; 8 Institute for Medical and Human Genetics, Charité Universitätsmedizin Berlin, Berlin, Germany; 9 Institute of Human Genetics, University Hospitals Schleswig-Holstein, University of Lübeck and Kiel University, 23562 Lübeck, Kiel, Germany; 10 DZHK (German Centre for Cardiovascular Research), Partner Site Hamburg/Lübeck/Kiel, Lübeck, Germany; 11 Center of Excellence for Neuropsychiatry, Vincent van Gogh Institute for Psychiatry, Venray, the Netherlands; 12 Department of Cognitive Neuroscience, Radboudumc, Nijmegen, the Netherlands

**Keywords:** SOX3, X-linked split-hand/foot malformation, SHFM2, Structural variation, Whole genome sequencing, Optical genome mapping

## Abstract

Split-hand/foot malformation (SHFM) is a congenital limb defect most typically presenting with median clefts in hands and/or feet, that can occur in a syndromic context as well as in isolated form. SHFM is caused by failure to maintain normal apical ectodermal ridge function during limb development. Although several genes and contiguous gene syndromes are implicated in the monogenic etiology of isolated SHFM, the disorder remains genetically unexplained for many families and associated genetic loci. We describe a family with isolated X-linked SHFM, for which the causative variant could be detected after a diagnostic journey of 20 years. We combined well-established approaches including microarray-based copy number variant analysis and fluorescence *in situ* hybridization coupled with optical genome mapping and whole genome sequencing. This strategy identified a complex structural variant (SV) comprising a 165-kb gain of 15q26.3 material ([GRCh37/hg19] chr15:99795320-99960362dup) inserted in inverted position at the site of a 38-kb deletion on Xq27.1 ([GRCh37/hg19] chrX:139481061-139518989del). *In silico* analysis suggested that the SV disrupts the regulatory framework on the X chromosome and may lead to *SOX3* misexpression. We hypothesize that *SOX3* dysregulation in the developing limb disturbed the fine balance between morphogens required for maintaining AER function, resulting in SHFM in this family.

## Teaser

After a diagnostic journey of 20 years, a combination of genomics technologies detected the causative SV in a family with X-linked split-hand/foot malformation. The SV comprises insertion of 15q26.3 material on Xq27.1, disrupting the regulatory framework on the X chromosome and most likely leading to *SOX3* misexpression in the developing limb.

Split-hand/foot malformation (SHFM) refers to a heterogeneous group of rare congenital limb anomalies, characterized by median clefts in hands and/or feet, syndactyly, and aplasia or hypoplasia of the metacarpal, metatarsal, and phalangeal bones, mostly affecting the central rays.^1^^,^^2^ It arises during embryonic development as a result of failure to maintain normal function of the apical ectodermal ridge (AER), a critical signaling structure that directs morphogenesis of the developing limb along the proximal-distal axis.^3^^,^^4^^,^^5^^,^^6^^,^^7^ SHFM is a rare disease with a prevalence of 1 per 90,000 live births.^2^ The severity of clinical features varies substantially and ranges from syndactyly in mildly affected individuals to monodactyly or aphalangia in its most severe forms.^1^ This clinical variability can be observed between members of the same family but even between limbs of a single affected individual.^1^^,^^6^

SHFM can be observed in a syndromic context as well as in isolated (non-syndromic) form. To date, OMIM describes over 50 syndromes that involve SHFM, with a range of associated (congenital) anomalies, including neurodevelopmental delay, growth retardation, hearing loss, and craniofacial, ectodermal, and internal organ abnormalities.^2^^,^^8^^,^^9^ For the non-syndromic forms, various clinical criteria have been proposed to assist in its classification, which depend on anatomical and radiographic findings, such as position of the cleft and thumb web, unilateral or bilateral occurrence, involvement of the long bones, and clinical severity.^1^ Based on the associated genetic locus, 12 distinct forms of non-syndromic SHFM can be recognized, with an autosomal dominant, autosomal recessive or X-linked mode of inheritance, often showing variable expressivity and incomplete penetrance.^2^^,^^10^ Contiguous gene syndromes have been implicated in the etiology of SHFM, and for five of these 12 loci, the associated genes have been identified.^2^ These include *DLX5* and *DLX6* in SHFM1 (MIM#183600 and MIM#220600), *TP63* in SHFM4 (MIM#605289), *WNT10B* in SHFM6 (MIM#225300), *ZAK* in split-foot malformation with mesoaxial polydactyly (SFMMP; MIM#616890), and *EPS15L1* in SHFM8 (Table 1).^2^^,^^11^ Additionally, a combinatorial effect of ectopic misexpression of multiple genes was shown in the etiology of SHFM3.^12^

**Table 1:** Genetic aberrations in 12 non-syndromic forms of SHFM

Type	MIM/PMID	Locus	Inheritance	Genetic aberrations	Causative gene
SHFM1	#183600	7q21.2q21.3	AD	Duplication/deletion/rearrangement involving *DLX5, DLX6, DSS1* or possible regulatory elements; SNVs/indels of *DLX5* and *DLX6*	*DLX5, DLX6*
SHFM1D	#220600	7q21	AR	SNVs/indels in *DLX5*	*DLX5*
SHFM2	%313350	Xq26.3	XL	Unknown (linkage between DXS1114 and DXS1192, chrX:133,295,286-138,368,235)	Unknown
SHFM3	#246560	10q24	AD	(Micro)duplications and complex rearrangements, including *FGF8, LBX1, BTRC, POLL* and *FBXW4* (=*DACTYLIN*)	Unknown
SHFM4	#605289	3q28	AD	SNVs in *TP63*	*TP63*
SHFM5	%606708	2q31	AD	Unknown, possibly haploinsufficiency of HOXD gene cluster	Unknown
SHFM6	#225300	12q13.12	AR	SNVs/indels in *WNT10B*	*WNT10B*
SFMMP	#616890	2q31.1	AR	Intragenic deletions or SNVs of *ZAK*	*ZAK*
SHFM8	PMID:29023680 PMID:32021595	19p13.11	AR	Indel in *EPS15L1*	*EPS15L1*
		8q21.11q22.3	AR	Unknown	Unknown
SHFLD1	%119100	1q42.2q43	AD	Unknown	Unknown
SHFLD2	%610685	6q14.1	AD	Unknown	Unknown
SHFLD3	#612576	17p13.3	AD	(Micro)duplications involving *BHLHA9*	Unknown

AD, autosomal dominant; AR, autosomal recessive; XL, X-linked.

Finding genetic causes for SHFM is complicated by the rarity of the phenotype, the large number of morphogens associated with limb development, their complex interactions, including intertwining of signaling pathways acting in different spatial dimensions, and the presumed involvement of regulatory elements.^6^^,^^13^ SHFM2 (MIM%313350) is the only mapped form of non-syndromic SHFM with X-linked inheritance.^14^^,^^15^^,^^16^^,^^17^ Although X-linked inheritance for SHFM was already suggested in 1978,^17^ SHFM2 is merely based on a single, large consanguineous family reported first in 1987.^14^ In this family, 36 individuals in seven generations were affected by monodactyly or split-hand and split-foot, with full expression of the trait in hemizygous males and presumed homozygous females, whereas heterozygous females were either unaffected or showed milder phenotypes.^14^ Linkage analysis in this large family defined a 5.1 Mb region on Xq26.3 (between DXS1114 and DXS1192, (GRCh37/hg19) chrX:133,295,286–138,368,235).^15^^,^^16^ The exons and exon-intron boundaries of 19 candidate genes in the linkage region were sequenced, but no relevant variants were identified,^16^ suggesting that regulatory elements must be at play in the pathophysiology of SHFM2.

Here, we report on a family in which five individuals are affected by non-syndromic SHFM, consistent with an X-linked inheritance pattern. We applied a combined approach of microarray-based copy number variant (CNV) analysis, fluorescence *in situ* hybridization (FISH), optical genome mapping (OGM), and whole genome sequencing (WGS). The combination of these technologies enabled us to identify a complex structural variant (SV), consisting of a 165-kb duplicated fragment originating from chromosome 15, to be inserted in inverted orientation at the site of a 38-kb deletion on the X chromosome near the previously described SHFM2 locus and *SOX3*. We hypothesize that perturbations of regulatory elements lead to dysregulated *SOX3* expression affecting AER maintenance in the limb bud and causing SHFM in this family.

**Figure 1:**
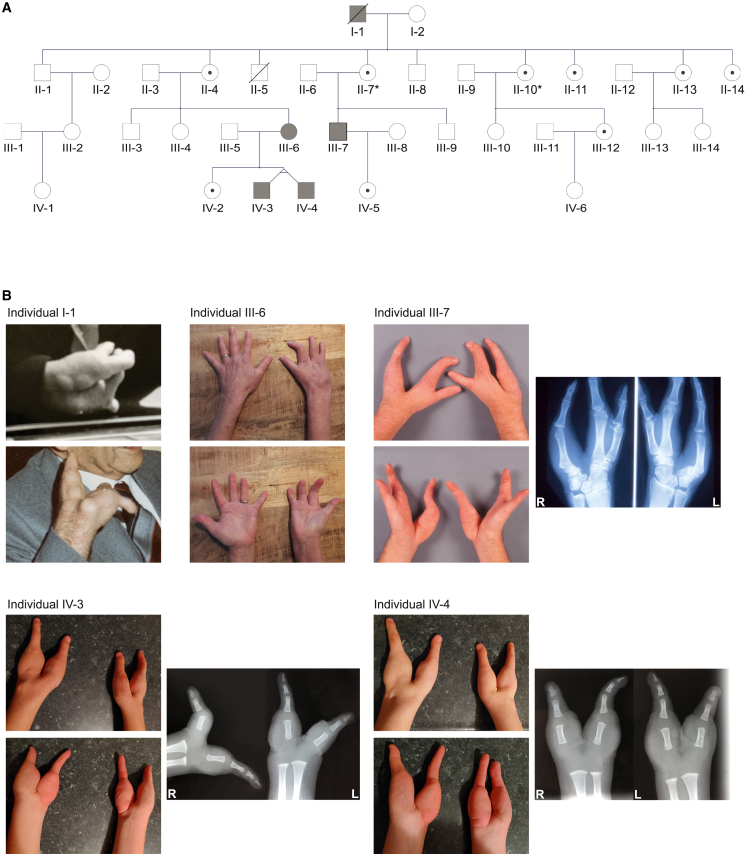
A family with X-linked split-hand malformation (A) Pedigree of the family showing five affected individuals (marked in gray) in the first, third, and fourth generation, and unaffected carriers (indicated by a dot) in the second, third, and fourth generation, consistent with an X-linked inheritance pattern. Radiographic feet abnormalities are indicated by an asterisk. (B) Photographs of upper limbs of individuals I-1, III-6, III-7, IV-3, and IV-4. Individual I-1 exhibited bilateral V-shaped hands with median clefts. Individual III-6 had right-sided split-hand with four fingers on the affected hand. Individual III-7 showed bilateral split-hand malformation, with abnormal carpal bone and absence of metacarpal and phalangeal bones of the third and fourth rays as seen on radiography. Monozygotic twins IV-3 and IV-4 are affected by bilateral bidactylous split-hand, characterized by deep V-shaped median clefts. Radiography of the hands at age 1 year and 9 months showed age-related absence of calcification of the carpal bones (therefore, the formation cannot be assessed based on these images) and complete absence of metacarpals and phalanges of the second, third, and fourth digits. The thumb consisted of a normal metacarpal and proximal phalangeal bone with a bifid distal phalangeal bone, and the fifth ray showed normal metacarpal and triphalangeal formation positioned in abnormal ulnar rotation.

Individuals from the reported family first received genetic counseling at a university medical center in the Netherlands over 20 years ago, and were followed up at low frequency thenceforth. In this four-generation kindred (Figure 1A), a total of five individuals were affected by congenital unilateral or bilateral non-syndromic split-hand malformation, but without clinical abnormalities of the feet. Evaluation of the pedigree indicated an X-linked mode of inheritance, or less likely, autosomal dominant inheritance with reduced penetrance. The Radboudumc review board approved the study (2019–5554) and affected individuals provided written informed consent.

Individual I-1 was a male affected by bilateral split-hand malformation (Figure 1B), who deceased at 82 years of age. He had three sons and six daughters from a non-consanguineous relationship, all of whom were clinically unaffected. Five of his nine children had offspring, but only his two oldest daughters (individual II-4 and II-7) had offspring with symptoms fitting the SHFM spectrum. Individual III-6, the 52-year-old daughter of individual II-4, showed a mild phenotype consisting of unilateral oligodactyly. She presented with four fingers in cleft-shape on the right hand (Figure 1B), requiring surgical intervention at age 18 months. Her brother (III-3), sister (III-4), and daughter (IV-2) were unaffected, but both her monozygotic twin sons (individual IV-3 and IV-4) exhibited bilateral split-hand malformation. The 22-year-old twins had very similarly affected hands characterized by absence of the three central digits, leading to a deep median cleft between the first and fifth rays, with the fifth ray in ulnar rotation. Radiography of hands in the posterior-anterior direction at age 1 year and 9 months showed a deep V-shaped cleft with complete absence of calcification of the carpals, as well as complete absence of metacarpals and phalanges of the second, third, and fourth rays. The thumb consisted of a normal metacarpal and proximal phalangeal bone, with a bifid top phalanx. The fifth ray comprised normal metacarpal, and normal proximal, mid and end phalangeal bones, positioned in abnormal ulnar rotation (Figure 1B). As this ulnar rotation interfered with grip and pinch function of hands, and herewith development of fine motor skills, the twins underwent a rotational osteotomy at the base of the fifth metacarpal of the right (dominant) hand at 2 years and 9 months to improve functional anatomy of the hand. They received hand therapy of a specialized occupational therapist. At age 3 years and 10 months, they could perform fine motor skills at a slow but age-appropriate level. Besides a diagnosis of attention-deficit/hyperactivity disorder in both, they had a normal development and there were no additional congenital abnormalities or medical problems. Their 50-year-old second cousin, individual III-7, son of individual II-7, showed a phenotype of bilateral split-hand malformation with absence of the third and fourth rays, including absence of metacarpal and phalangeal bones, as well as abnormalities of the carpal bones (large scaphoid, fused trapezoid and trapezium, absent capitate, rotated hamate, small triquetrum, pisiform and lunate), as seen on radiography (Figure 1B). His feet showed mild bilateral cutaneous 2-3-syndactyly (Figure S1). There were no other congenital anomalies or medical problems, and his brother (III-9) and daughter (IV-5) were unaffected. To understand segregation of the trait in this family, individuals II-1, II-4, II-7, II-10, II-11, III-4, and III-9 were screened for subclinical hand and feet abnormalities by radiography. For individual II-7, this showed bony coalition of talus and navicular bone and shortened second metatarsal bone of the right foot. Individual II-10 had bilateral shortened second metatarsal bones. All other imaging results were reported normal.

**Figure 2:**
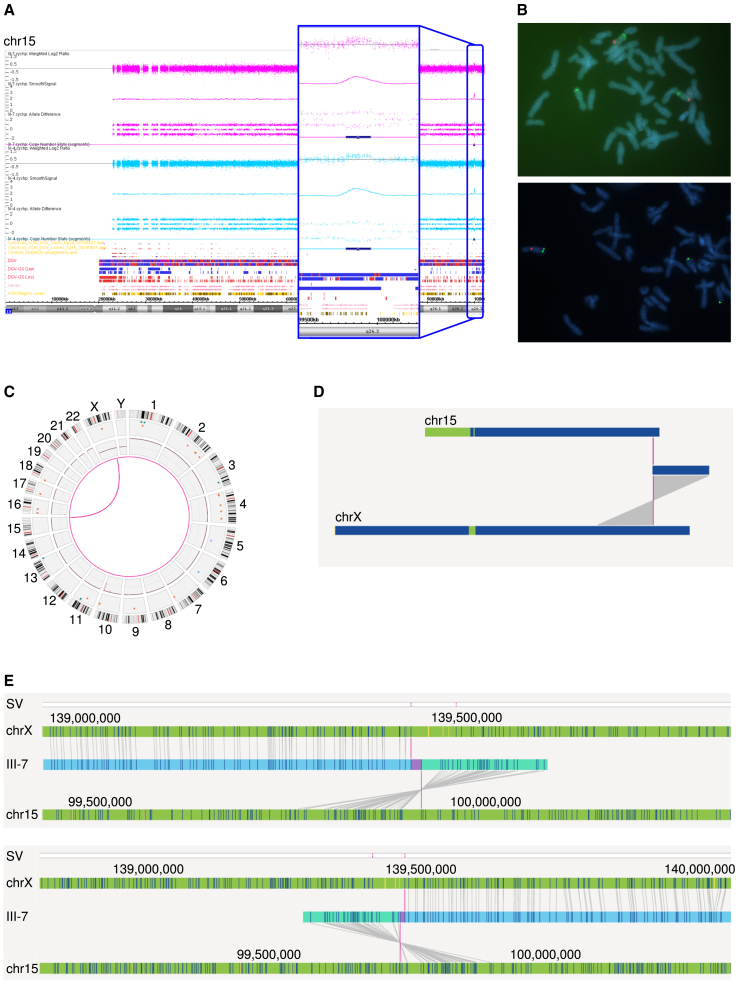
Microarray-based CNV analysis, FISH, and OGM detected an inverted 15q26.3 gain inserted in Xq27.1 (A) Microarray-based CNV analysis of individual III-7 and IV-4 shows a 15q26.3 gain of ∼161 kb. (B) FISH experiments show this 15q26.3 gain is inserted in the long arm of the X chromosome. Upper image: the centromeres of both chromosomes 15 (probe CEP 15, Vysis) are labeled in red. In green, probe RP11-668p3 (Empire Genomics) marks the duplicated region that is present on both chromosomes 15 with a third signal on Xq27, pinpointing that the gain of chromosome 15 is inserted into the distal long arm of the X chromosome. Lower image: metaphase FISH results with in red the centromere of chromosome X (probe CEP-X, Vysis) and in green probe RP11-668p3, that is present on both chromosomes 15 and again showing a third signal on the X chromosome. (C) Circosplot showing chromosomes X and 15 connected with a pink line, representing a translocation event. (D) Whole chromosome view from OGM, indicating a translocation between chromosome 15 and the X chromosome with the pink line, t(X; 15)t(q27.1; q26.3). (E) OGM results from individual III-7 shows the 15q26.3 gain inserts in an inverted fashion on the X chromosome. The upper image illustrates the left breakpoint, the lower image illustrates the right breakpoint. In both these images, the upper green bar indicates the X chromosome, the middle blue bar indicates the genome of individual III-7, and the lower bar indicates chromosome 15. Each of the lines in these bars represents the fluorescent labels targeting a 6-nucleotide motif that occurs randomly throughout the genome. The lines connecting labels found in individual III-7 with labels on the X chromosome and on chromosome 15 indicate the software recognizes these labels are the same.

Over the course of the years, genetic testing in this family included chromosome X-specific exome sequencing, and targeted sequencing of *TP63*, associated with autosomal dominant SHFM4 with reports of remarkable non-penetrance,^4^^,^^10^ but neither identified a causative variant. Additionally, linkage analysis failed to identify a locus with a significant logarithm of odds (LOD) score, although analysis with markers from the SHFM2 locus had revealed that linkage in the present family would be compatible with the SHFM2 locus identified in the previously published family.^14^^,^^15^^,^^16^ Because of the family’s ongoing search for a genetic diagnosis and CNVs being implicated in several forms of SHFM, we used DNA isolated from blood of individual III-7 for microarray-based CNV analysis (Affymetrix CytoScan HD 2.6M), using routine diagnostic procedures^18^ and reporting based on genome build GRCh37/hg19. This analysis identified two rare CNVs, of which the first was an intragenic deletion of 175 kb in 10q26.2, arr [GRCh37/hg19] 10q26.2(129,012,678-129,186,196)x1, including *DOCK1*. The second CNV was an interstitial gain of 161 kb in 15q26.3, arr [GRCh37/hg19] 15q26.3(99,796,482-99,957,320)x3, encompassing the gene *LRRC28* and pseudogene *HSP90B2P.* These CNVs were neither reported before by our diagnostic laboratory, nor in online CNV databases of healthy or affected individuals.^19^^,^^20^^,^^21^ In addition, no disease-gene associations were known for *DOCK1, LRRC28,* or *HSP90B2P* in OMIM.^8^^,^^9^ Therefore, both CNVs were classified as variants of uncertain significance. To gain further insights in the clinical relevance of these CNVs, we continued with array-based CNV analysis in an additional affected relative (individual IV-4), who was, together with his monozygotic twin brother, the most distant affected individual in the pedigree (separated by five meiotic cell divisions from III-7). In individual IV-4, the 10q26.2 deletion was not present, but the 15q26.3 gain as found in individual III-7 was detected (Figure 2A). Based on this observation and the pedigree information, his affected twin brother (individual IV-3), affected mother (individual III-6), and affected great-grandfather (individual I-1) are likely carriers of the 15q26.3 gain, which herewith segregates with the SHFM phenotype. However, an aberration on one of the autosomes would not explain the classic X-linked inheritance in this family, as evaluated from the absence of SHFM in the entire second generation, although there are at least two obligate female carriers of the 15q26.3 gain (individual II-4 and II-7), together with the observation of a milder phenotype in the only affected female (individual III-6) compared with her male counterparts. We thus hypothesized that the observed 15q26.3 gain could be part of a more complex SV, possibly involving the X chromosome.

We continued with karyotyping and metaphase FISH experiments in duplicate on cultured Epstein-Barr virus immortalized lymphoblastoid cells (EBV-LCLs) of affected male individual III-7, using RP11-668P3 (Empire Genomics, Williamsville, NY, USA) as a region-specific probe for 15q26.3 and probe CEP-15 (Vysis, Abbott, Abbott Park, IL, USA) for the centromere of chromosome 15 or probe CEP-X (Vysis, Abbott, Abbott Park, IL, USA) for the centromere of chromosome X. Conventional karyotyping showed a normal male karyotype as the copy number gain of 15q26.3 is smaller than the detection limit of G-banding (5–10 Mb). FISH, however, showed an abnormal pattern with the duplicated segment of 15q26.3 resulting in a third signal on the long arm of the X chromosome (Figure 2B). The exact locus of insertion of the duplicated fragment of chromosome 15 into chromosome X could not be determined, but based on the FISH experiments, this was most probably at Xq27 (46,der(X)ins(X; 15) (q2?7; q26.3q26.3),Y. ish der(X) (q2?7) (RP11-668P3+)).

Although FISH confirmed our hypothesis of a structural chromosome rearrangement involving not only chromosome 15 but also the X chromosome, it lacked the resolution to elucidate the exact genomic architecture underlying the variant. Therefore, we continued with a two-pronged approach consisting of parallel OGM and WGS to gain insights in the breakpoints and orientation of the individual elements of the SV. OGM technology (Bionano Genomics) generates ultra-long DNA fragments (≥150 kb) with fluorescent labels targeting a 6-nucleotide motif occurring non-randomly throughout the genome, resulting in a *de novo* genome assembly with "barcode-like" visualization of the genome of interest, and a more than 10,000-fold higher resolution than conventional karyotyping.^22^^,^^23^ Comparison of the occurrence of this 6-nucleotide motif between the genome of interest and a reference allows for detection of CNVs and (complex) SVs.^22^^,^^23^ We isolated ultra-high molecular weight DNA from EBV-LCLs of individual III-7, followed by the previously published OGM workflow,^22^^,^^23^ including *de novo* genome assembly and variant calling of CNVs and SVs. We obtained an N50 molecule length (of molecules larger than 150 kb) of 0.321 Mbp and a map rate of 85.9%, resulting in an effective coverage of 224-fold.^22^ Variants were prioritized as previously described.^22^ OGM readily revealed the variant of interest in more intricate detail: the gain originating from chromosome 15 ([GRCh37/hg19] chr15:99795709-99959476) inserted in an inverted fashion at the Xq27.1 locus between positions [GRCh37/hg19] chrX:139468438 and chrX:139527176 (Figures 2C–2E and S2, and Table S1A). Additionally, visual inspection of output from the CNV algorithm suggested the presence of a small deletion on Xq27.1 at the locus of the insertion, although not detected by the CNV calling software.

**Figure 3:**
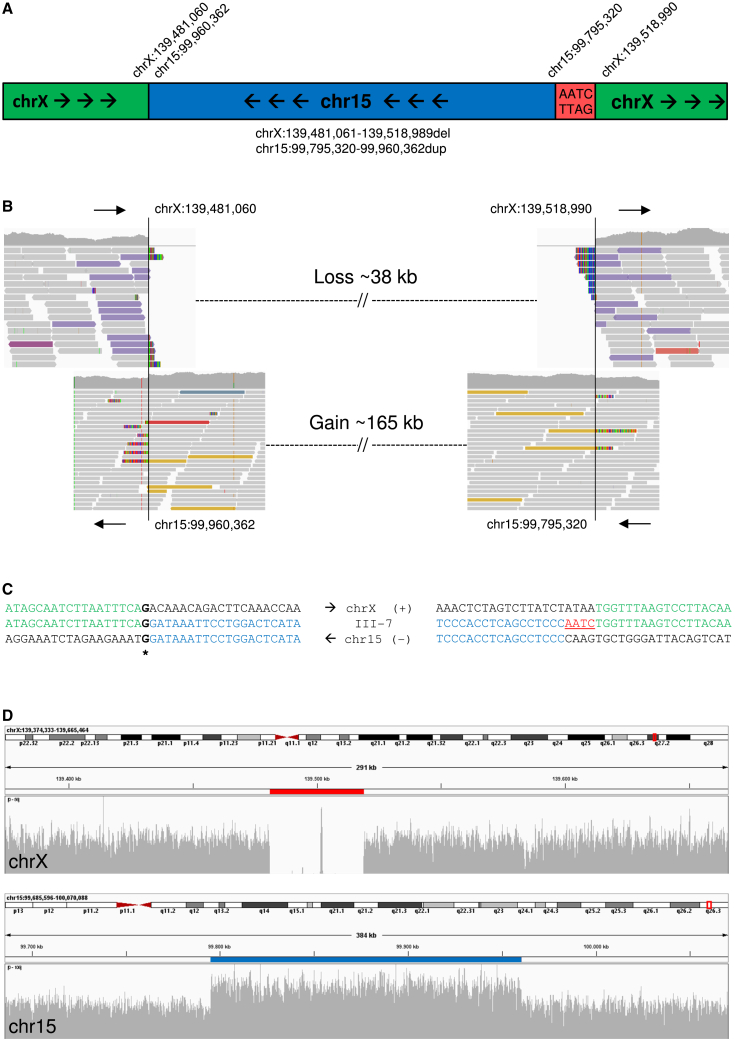
Detection of SV breakpoints at single nucleotide resolution with WGS (A) Schematic representation of the structural variant, comprising an inverted 165-kb gain from 15q26.3 inserted at the site of a 38-kb deletion on Xq27.1. (B) Visualization of alignment of reads from WGS in IGV. The proximal (left) breakpoint on the X chromosome attaches to the distal end of the duplicated fragment of chromosome 15, whereas the distal (right) breakpoint on the X chromosome is connected with the proximal end of the duplicated fragment of chromosome 15. (C) Sequence alignment at both breakpoints, showing insertion of four nucleotides of unknown origin at the distal breakpoint on the X chromosome. (D) Coverage data obtained from WGS shows a 38-kb deletion on the X chromosome (upper image, red line) and a 165-kb duplication on chromosome 15 (lower image, blue line).

WGS (BGISeq500, PE100) was performed in parallel to OGM on DNA derived from blood of individual III-7. Prioritization of SNVs/indels, CNVs and SVs from WGS data did not reveal any other compelling variants,^24^^,^^25^ but allowed us to refine the breakpoints of the SV at single nucleotide resolution. We obtained a mean coverage of 40-fold, and data were interpreted with a targeted approach, prioritizing CNVs (Control-FREEC^26^ and Canvas^27^) and SVs (Manta^28^) on 15q26.3 and Xq27.1 using an ANNOVAR-based^29^ annotation pipeline. Both CNV calling algorithms identified the rare 15q26.3 gain observed in previous experiments (Tables S1B and S1C). Additionally, both tools detected a rare ∼38 kb Xq27.1 deletion (Tables S1B and S1C). Further analysis of the Manta output suggested the duplicated fragment of 15q26.3 to be inserted in inverted orientation at the locus of the Xq27.1 deletion, with inclusion of four nucleotides of unknown origin at the right breakpoint on the X chromosome (Table S1D). Visual inspection of WGS data in the Integrative Genomics Viewer (IGV)^30^ (Figures 3A–3D, S3, and S4) and analysis of breakpoints using BLAT on DNA^31^ confirmed the 165-kb duplicated fragment from chromosome 15 ([GRCh37/hg19] chr15:99795320-99960362dup) inserted at the site of a 38-kb deletion ([GRCh37/hg19] chrX:139481061-139518989del) on the X chromosome (Figure 3A). Both breakpoints were confirmed by PCR and Sanger sequencing. Segregation analysis by breakpoint-spanning PCRs, including all family members for whom DNA was available (23 individuals), confirmed the presence of the SV in all affected individuals (III-6, III-7, IV-3, and IV-4) and in all putative carriers (II-4 and II-7). In addition, it revealed seven additional clinically unaffected female carriers (II-10, II-11, II-13, II-14, III-12, IV-2, IV-5; Figure S5). From these data, we concluded that the SV segregates with the SHFM phenotype (Figure 1A) and that expression of clinical features is consistent with an X-linked inheritance pattern.

Of all female carriers, only individual III-6 showed a mild unilateral split-hand malformation, whereas the other nine did not. Additionally, individual II-7 and II-10 exhibited mild subclinical radiographic foot abnormalities, although it remains uncertain whether these foot abnormalities are implicated in SHFM2 or occur randomly. We hypothesized that differences in X-inactivation could play a role in phenotypic variability between female carriers and continued with X-inactivation studies by quantifying methylation of the human androgen receptor locus including all 10 confirmed female carriers (II-4, II-7, II-10, II-11, II-13, II-14, III-6, III-12, IV-2, and IV-5). However, this analysis did not yield any conclusive results to understand the difference in phenotypic presentation among female carriers (Figure S6; supplemental information).

Collectively, the proximity of the here described SV to the known SHFM2 locus, the absence of variation in this locus in control populations, and the segregation pattern observed in the family, strongly suggest that this variant is causative for the SHFM in this family. We therefore pursued further biological and functional understanding of this variant by focusing on three aspects, being (1) the genomic contents of the duplicated fragment of chromosome 15, (2) the genomic contents deleted on chromosome X, and (3) the disruption of regulatory context by the insertion of chromosome 15 material into the X chromosome.

The duplicated segment of chromosome 15 contains part of *LRRC28* and pseudogene *HSP90B2P*. Despite *LRRC28* not being triplosensitive (pTriplo 0.07),^32^ and the duplicated protein-coding sequencing only involving exons 2–10, we first hypothesized that this duplication could play a role in SHFM pathophysiology in this family (Figure S7A). *LRRC28* is ubiquitously expressed and encodes Leucine Rich Repeat Containing protein 28 (LRRC28), characterized by Leucine Rich Repeats (LRRs). Although its exact functions are largely uncharacterized, LRRC28 was reported to be involved in RAS-mediated signaling.^33^^,^^34^ We measured *LRRC28* expression with quantitative real-time PCR experiments on RNA isolated from cultured EBV-LCLs of individuals III-6, III-7, IV-3, and IV-4, alongside three male and three female healthy unrelated controls, and found that expression of *LRRC28* is not affected in individuals carrying the variant (Figures S7B and S7C), suggesting that partial *LRRC28* duplication is, as anticipated, not the underlying molecular cause of SHFM.

The 38-kb deletion on Xq27.1 does not contain any (protein-coding) genes. Interestingly, Xq27.1 is prone to structural variation, and insertions occurring at or near a quasi-palindromic sequence (ChrX:139,502,865–139,503,044) are associated with nine other distinct disease phenotypes.^35^ For two of these disease phenotypes, the underlying pathophysiological mechanism is shown to be dysregulation of a nearby gene, being *FGF13*^36^ and *SOX3*,^37^ respectively. We thus progressed to our hypothesis that the SV disrupts the genomic context, spatial organization and possibly regulation of genes in close proximity to the breakpoints. Assessing the region involved (300 kb up- and downstream of breakpoints) identified the protein-coding gene *SOX3* and two non-coding genes of which little is known, including the small nuclear RNA *U7* (*LOC124905265*; RF00066) and the long non-coding RNA *LINC00632.*^38^ *SOX3*, located 67 kb downstream of the SV breakpoint encodes SRY-Box Transcription Factor 3 (SOX3), a member of the SOX family of transcription factors, which includes important regulators of cell fate during embryonic development.^39^ *SOX3* is predominantly expressed in the fetal brain and spinal cord,^40^ where it is implicated in central nervous system development,^41^^,^^42^^,^^43^^,^^44^ by regulating gene expression in neural progenitor cells.^44^ Duplication of *SOX3* has been associated with intellectual disability with isolated growth hormone deficiency (MIM#300123)^45^ and panhypopituitarism (MIM#312000). Additionally, an SV located 67 kb downstream of *SOX3* is implicated in hypoparathyroidism,^46^ and *SOX3* dysregulation caused by insertion of chromosome 1 material at Xq26.3 is implicated in 46,XX male sex reversal (MIM#300833).^37^

**Figure 4:**
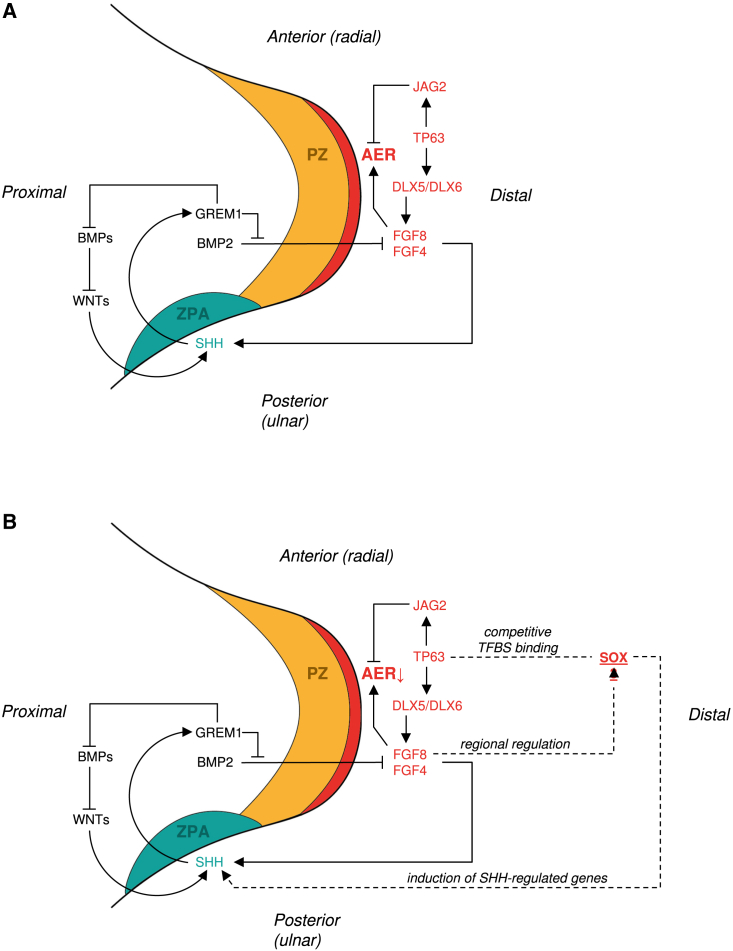
Misexpression of *SOX3* is hypothesized to disturb AER maintenance (A) Schematic representation of the developing limb bud in early limb formation with simplified representation of genes involved in AER maintenance. The AER in red orchestrates outgrowth of the limb along the proximal-distal axis. Mesenchymal cells in the progress zone (PZ) in orange stay in an undifferentiated state under AER control. The zone of polarizing activity (ZPA) in turquoise regulates development along the anterior-posterior (radial-ulnar) axis. Factors determining dorsal-ventral growth are not shown. Signaling pathways associated with polarizing activity along the three axes are intertwined. Patterning along the anterior-posterior axis is mediated by SHH, also required for integrity of the AER. Patterning along the proximal-distal axis is regulated by FGFs, including *FGF4* and *FGF8*, maintaining AER integrity. *TP63*, the gene underlying SHFM4, stimulates expression of *FGF8*, via induction of *DLX5* and *DLX6* (implicated in SHFM1), herewith contributing to AER maintenance. *TP63* can also negatively regulate AER maintenance via induction of *JAG2*. (B) We hypothesize that *SOX3* misexpression in the developing limb bud disturbs the balance between signaling molecules needed for AER maintenance, for example by competitive TFBS binding with TP63 or by induction of SHH-regulated genes.

*SOX3* is a plausible candidate in the etiology of SHFM2, as other members of the SOX family of transcription factors, such as *SOX5, SOX6, SOX8, SOX9,* and *SOX10*, are implicated in limb chondrogenesis.^47^ In addition, induced ectopic expression of *SOX3* in the limb bud alters expression patterns of Sonic Hedgehog (SHH)-regulated genes,^48^ and during early development, *SOX3* is co-expressed at the neural plate border with *TP63,*^49^ the gene implicated in SHFM4 (MIM#605289).^4^ SOX3 and TP63 share numerous transcription factor binding sites for neural plate genes, with TP63-dependent inhibition of SOX3 binding.^49^ Together, these proteins are thought to define the distinction between surface ectoderm and neuroectoderm.^49^ In the limb bud, TP63 is an important factor for AER maintenance, via regulation of *DLX5, DLX6,* and *FGF8* expression.^50^^,^^51^ *Tp63*^−/−^ mutant mice display defective AER maintenance, lack several limb components, and show reduced expression of *fgf8* in the limb bud,^52^ a gene involved in AER maintenance and thought to regulate regionalized expression of *SOX3* (Figure 4A).^53^

**Figure 5:**
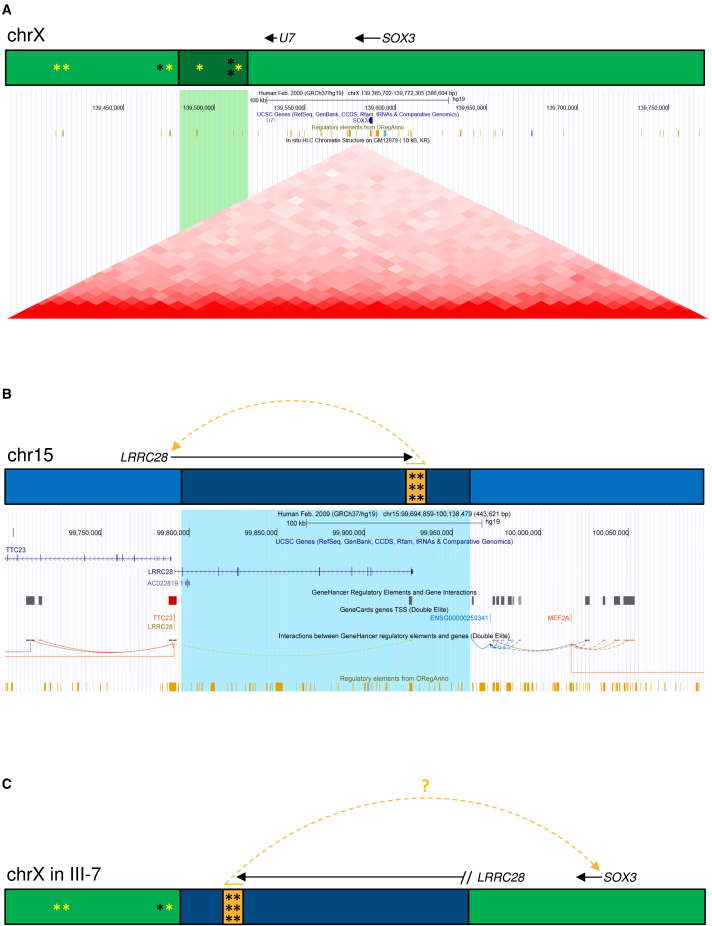
The SV is predicted to disturb regulation on the X chromosome (A) Schematic representation of genes and regulatory elements on the X chromosome (upper image), and screenshot from the UCSC genome browser with the 38-kb deletion on the X chromosome in green (lower image). The deleted fragment of the X chromosome (dark green in the schematic representation) is located ∼67 kb proximal of *SOX3* and contains four TFBSs (asterisk), of which two target *SOX3* (yellow asterisk; OREG1571551, TFBS of *FOXA1*; OREG1521521, TFBS of *ESR1*). In the 100-kb region proximal to the deleted fragment on the X chromosome, there are four TFBSs as well, of which three target *SOX3* (OREG1412659, TFBS of *E2F1*; OREG1412658 TFBS of *E2F1*; OREG1571552, TFBS of *FOXA1*). (B) Schematic representation of genes and regulatory elements on chromosome 15 (upper image), and screenshot from the UCSC genome browser with the 15q26.3 gain in blue (lower image). The duplicated 15q26.3 fragment (dark blue in the schematic representation) contains exon 2 to exon 10 of *LRRC28* and an enhancer (orange in the schematic representation), normally regulating *LRRC28* expression. This enhancer contains six TFBSs, associated with binding of *CTCF, HNF4A, FOSL2, JUND, JUNB,* and *SMARCA4*. (C) Schematic representation of regulatory elements affected by the SV. Two TFBSs targeting *SOX3* are deleted, the distance between three *SOX3*-targeting TFBS and *SOX3* is increased, and an enhancer is inserted on the X chromosome, which may retain its transcription-activation functions in the new genomic context. All these factors may affect expression of nearby genes, such as *SOX3.*

Taking this information together, we hypothesized that the SV interferes with tissue-specific and time-dependent regulation of *SOX3* expression, although it is also a possibility that genes even more distant from the breakpoints are dysregulated.^35^ Unfortunately, *SOX3* is neither expressed in any accessible tissue nor in cultured fibroblasts or EBV-LCLs, limiting the possibility to test this hypothesis. Additionally, it is uncertain whether measuring gene expression would accurately reflect the *in vivo* situation if not taking into account the relevant developmental time point and cell type. Regardless, given the observed *SOX3* misexpression in EBV-LCLs in 46,XX male sex reversal associated with an insertion near *SOX3,*^37^ we measured *SOX3* expression with quantitative real-time PCR experiments on RNA isolated from cultured EBV-LCLs of individuals III-6, III-7, IV-3, IV-4, and three male and three female healthy unrelated controls, also taking along *FGF13.*^36^ Expression levels of *SOX3* and *FGF13* in EBV-LCLs were too low in affected individuals as well as controls (data not shown), herewith not providing any further insights in our hypothesis. We therefore continued by *in silico* analysis reasoning that the SV would either disrupt topological associating domain (TAD) structures, and/or affect SOX3 transcription factor binding sites (TFBS). We found that the SV deletes four TFBSs on the X chromosome,^54^^,^^55^^,^^56^ two of which have *SOX3* as a target gene. Additionally, four TFBSs locate in a 100-kb region proximal to the locus of the inserted fragment on Xq27.1, and for three of these, *SOX3* is the target gene. Potentially, the SV affects *SOX3* expression as a result of loss of the TFBS and/or increased distance or disturbed genomic 3D organization between target gene *SOX3* and TFBS located proximal of the SV (Figures 5A and S8, and Table S2A). Assessing whether a similar SV is implicated in the etiology of SHFM2 in the previously published family^14^ would be an interesting future prospect, given that the linkage region from literature^15^^,^^16^ and the SV reported here are approximately 1.1 Mb apart but partly locate to the same TAD (Figure S8).

The duplicated 15q26.3 fragment inserted on the X chromosome also contains regulatory sequences, such as an enhancer (GH15J099385) that normally interacts with the *LRRC28* promoter located upstream on chromosome 15 (GH15J099248; Figures 5B and S9). This enhancer contains TFBS of *CTCF, HNF4A, FOSL2, JUND, JUNB,* and *SMARCA4* (Table S2B), of which *SMARCA4* is implicated in tissue-specific developmental gene regulation^57^ and limb development in mice, with abnormal AER morphology and defective hindlimb development associated with *SMARCA4* ablation (MGI:3606859).^58^ As enhancers are known for their functional autonomy, also when combined with heterologous promoters and genes,^59^ one could speculate that the inserted enhancer may retain its transcription-activating function in the new genomic context and as a result might cause *SOX3* misexpression (Figure 5C). Additionally, the duplicated region contains 10 candidate *cis*-Regulatory Elements that are active in the human developing limb (Table S3).^60^ Therefore, we hypothesize that misexpression of *SOX3* in the limb bud disturbs the fine balance required for AER maintenance as orchestrated by TP63 and FGF8, possibly resulting from competitive binding between SOX3 and TP63, or resulting from altered SHH-activity (Figure 4B),^5^^,^^6^^,^^7^^,^^48^^,^^50^^,^^51^^,^^53^^,^^61^^,^^62^ leading to SHFM in this family.

In summary, we describe a family with X-linked non-syndromic SHFM. By combining older but well-established approaches with innovative genomics technologies, we could delineate the exact genetic aberration underlying SHFM in this family. The phenotype is caused by a complex SV, comprising an inverted 165-kb 15q26.3 gain inserted at the site of a 38-kb Xq27.1 deletion. As the SV does not alter expression of the partly duplicated protein-coding gene *LRRC28*, we anticipate that the SV influences regulatory functions of the affected region on the X chromosome, including *SOX3* as a plausible candidate gene. Hence, we hypothesize that the SV causes misexpression of *SOX3* in the developing limb bud, resulting in failure to maintain AER function.

## Data Availability

Disclosure of the full (raw and annotated) datasets are restricted by the level of consent for data sharing provided by the participants. Requests for more detailed analyses and (partial) data access should be discussed with the corresponding author.

## Web resources

https://omim.org/.

http://www.oreganno.org/dump/.

https://screen.wenglab.org/.

## Supplemental information

Document S1. Figures S1–S9 and Materials and methods

Table S1. Overview of variant calls indicative of the SV derived from OGM and WGS data

Table S2. Overview of annotated human transcription factor binding sites affected by the SV

Table S3. Overview of candidate *cis*-Regulatory Elements in human embryonic limb tissues in the chromosome 15 gain

Document S2. Article plus supplemental information
