# Supplementary material for: A complex structural variant near SOX3 causes X-linked split-hand/foot malformation
Source: HGG Adv. 2023 Apr 25;4(3):100200. doi: 10.1016/j.xhgg.2023.100200 (PMC10196709; doi:10.1016/j.xhgg.2023.100200)
Supplement: Document S1. Figures S1–S9 and Materials and methods [file mmc1.pdf]

HGGA, Volume *4*

Supplemental information

A complex structural variant near *SOX3* causes X-linked split-hand/foot malformation

Elke de Boer, Carlo Marcelis, Kornelia Neveling, Ellen van Beusekom, Alexander Hoischen, Willemijn M. Klein, Nicole de Leeuw, Tuomo Mantere, Uirá S. Melo, Jeroen van Reeuwijk, Dominique Smeets, Malte Spielmann, Tjitske Kleefstra, Hans van Bokhoven, and Lisenka E.L.M. Vissers

Figure S1: Feet of individual III-7 showing mild cutaneous 2-3-syndactyly

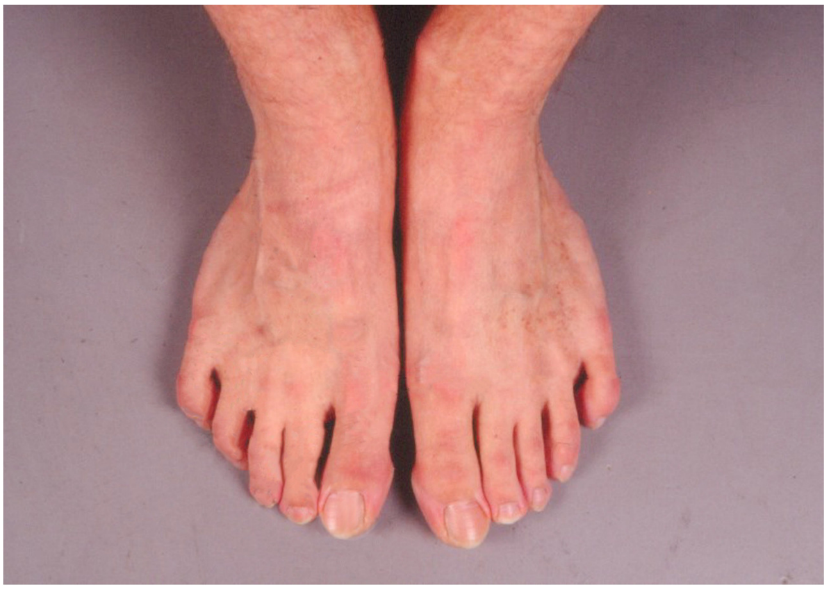

The feet of individual III-7 show mild cutaneous 2-3-syndactyly. Although syndactyly is common in SHFM, cutaneous 2-3-syndactyly of the toes is a non-specific clinical finding and relatively common in the general population.

Figure S2: OGM detects an inverted 15q26.3 gain inserted on Xq27.1

A.

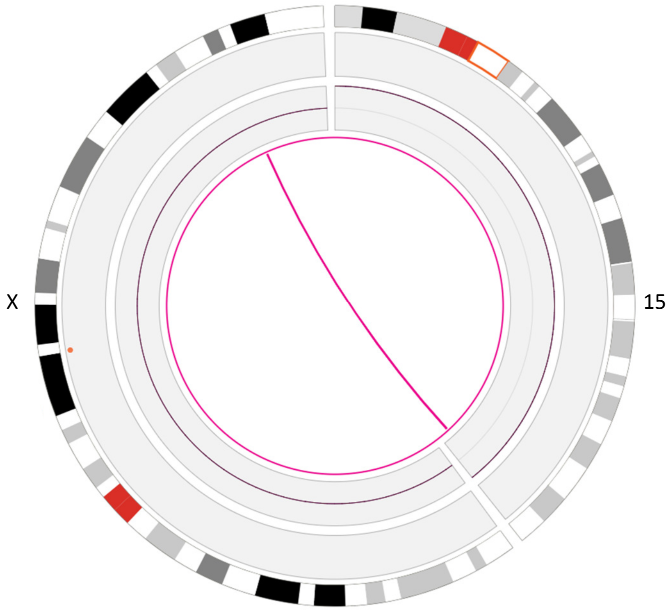

B.

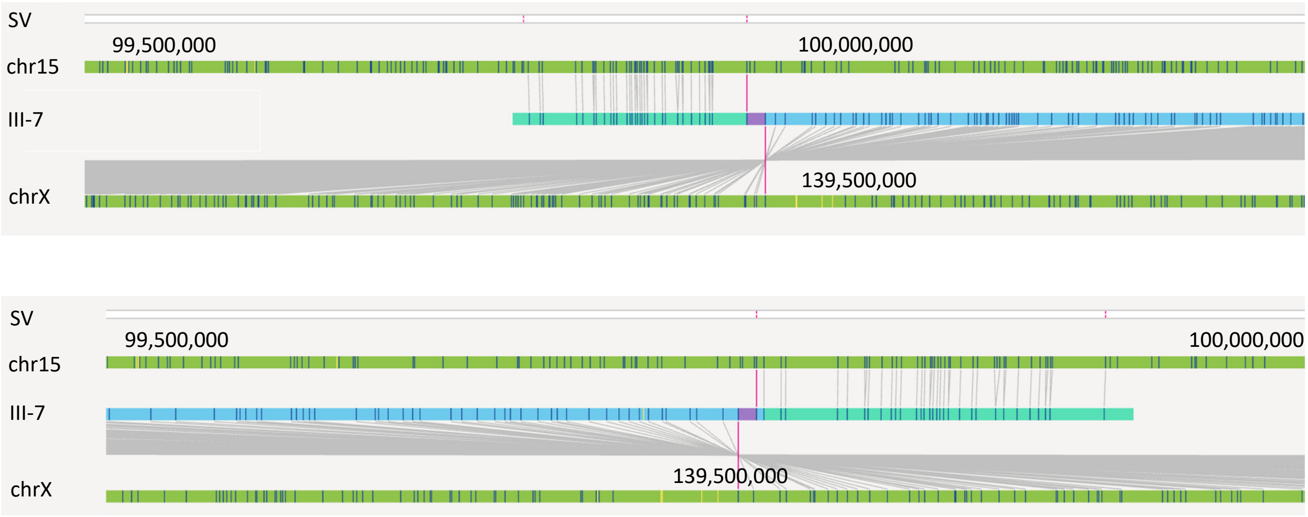

A) Circosplot showing a translocation between chromosome 15 and the X-chromosome. B) OGM results from individual III-7 indicates that the 15q26.3 gain inserts in an inverted fashion on the X-chromosome. The upper image illustrates the proximal breakpoint (distal end of gain from chromosome 15), the lower image illustrates the distal breakpoint (proximal end of gain from chromosome 15).

Figure S3: Visualization of the proximal breakpoint from WGS data

A.

```
→ Chromosome X (+)      GGATAGCAATCTTAATTTCAGACAAACAGACTTCAAACCAA
Individual III-7        GGATAGCAATCTTAATTTCAGGATAAATTCCTGGACTCATA
← Chromosome 15 (-)     CTAGGAAATCTAGAAGAAATGGATAAATTCCTGGACTCATA
```

B.

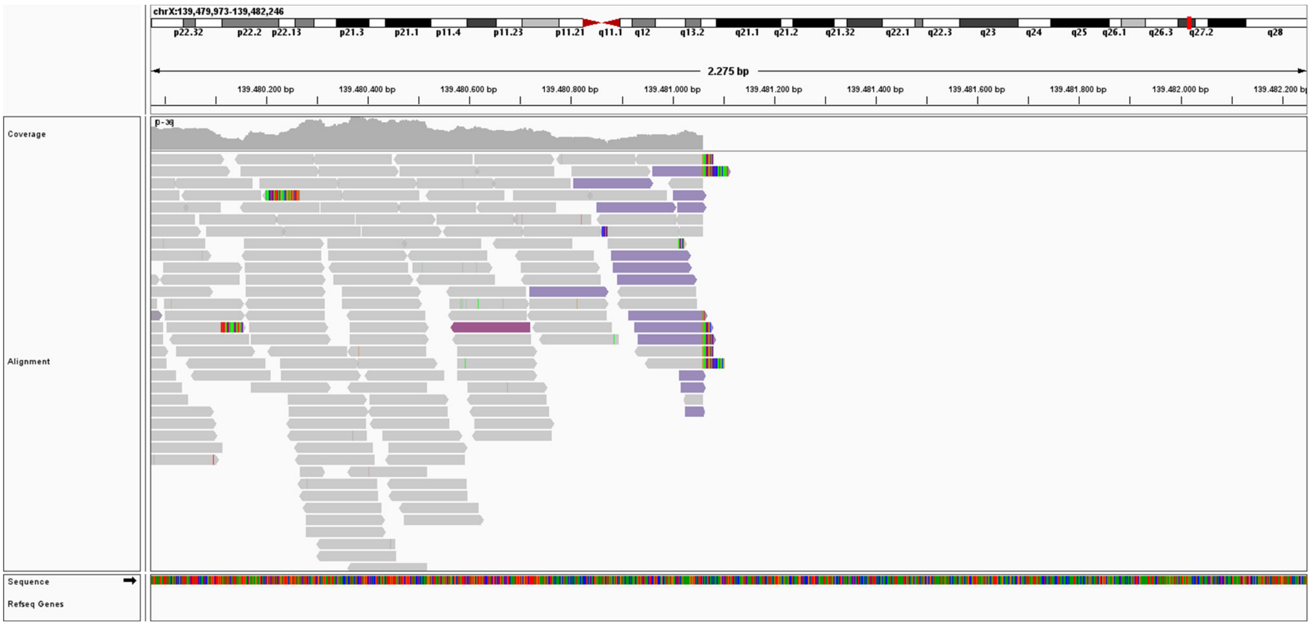

C.

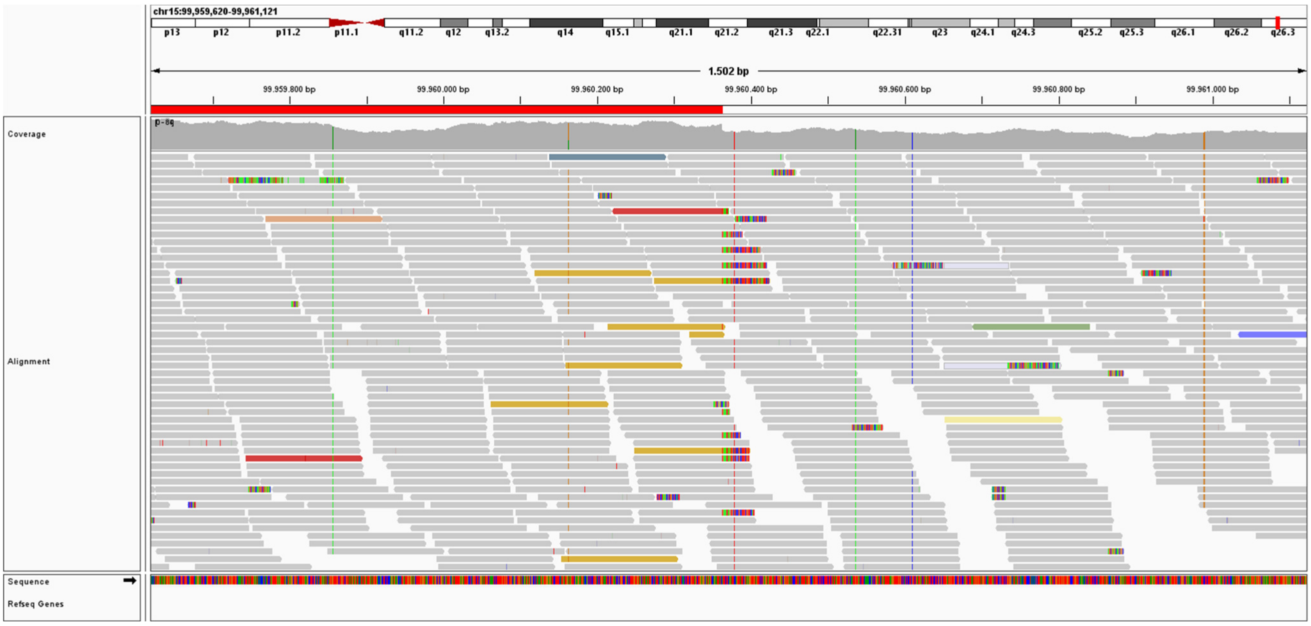

A) Alignment of the sequence at the proximal breakpoint. B) IGV screenshot of the proximal end of the deletion on the X-chromosome. C) IGV screenshot of the distal end of the duplication on chromosome 15.

Figure S4: Visualization of the distal breakpoint from WGS data

A.

```
→ Chromosome X (+)      GGAAACTCTAGTCTTATCTATAATGGTTTAAGTCCTTACAA
Individual III-7        CCTCCCACCTCAGCCTCCCAATCTGGTTTAAGTCCTTACAA
← Chromosome 15 (-)     CCTCCCACCTCAGCCTCCCCAAGTGCTGGGATTACAGTCAT
```

B.

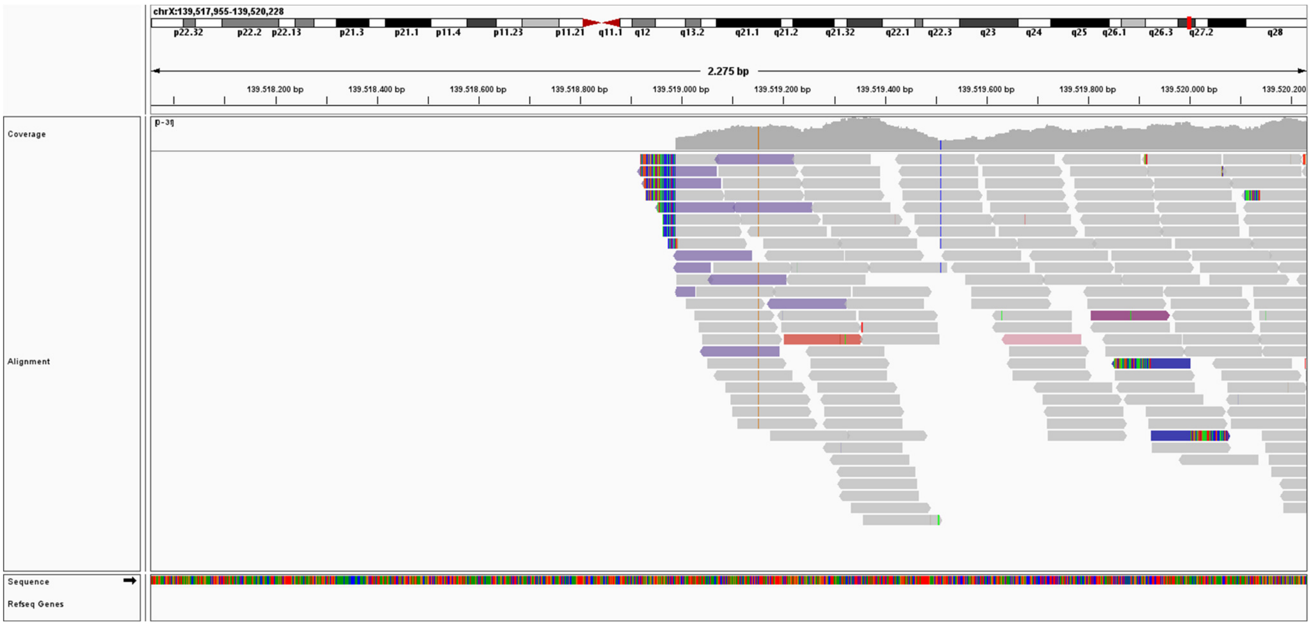

C.

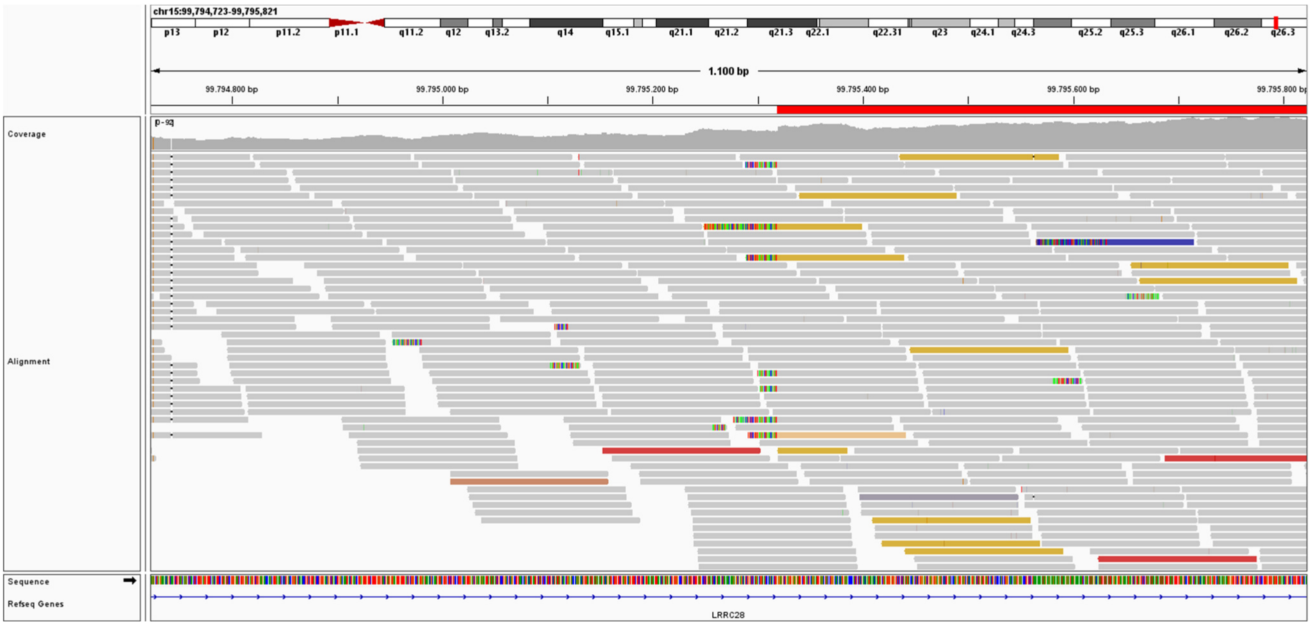

A) Alignment of the sequence at the distal breakpoint, including four nucleotides of unknown origin. B) IGV screenshot of the distal end of the deletion on the X-chromosome. C) IGV screenshot of the proximal end of the duplication on chromosome 15.

Figure S5: Validation and segregation of breakpoint junctions

A.

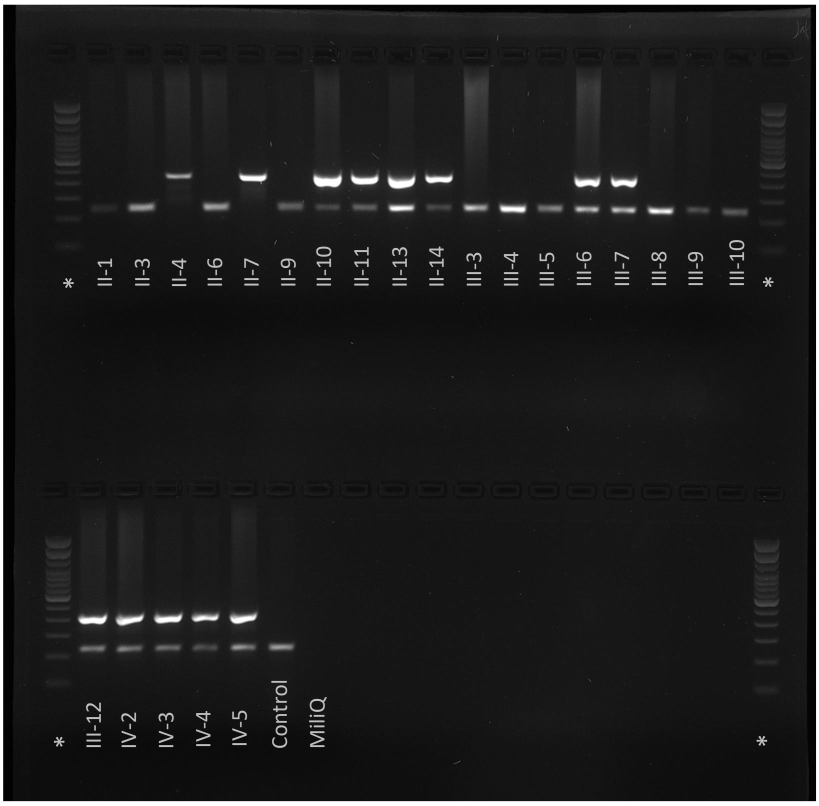

B.

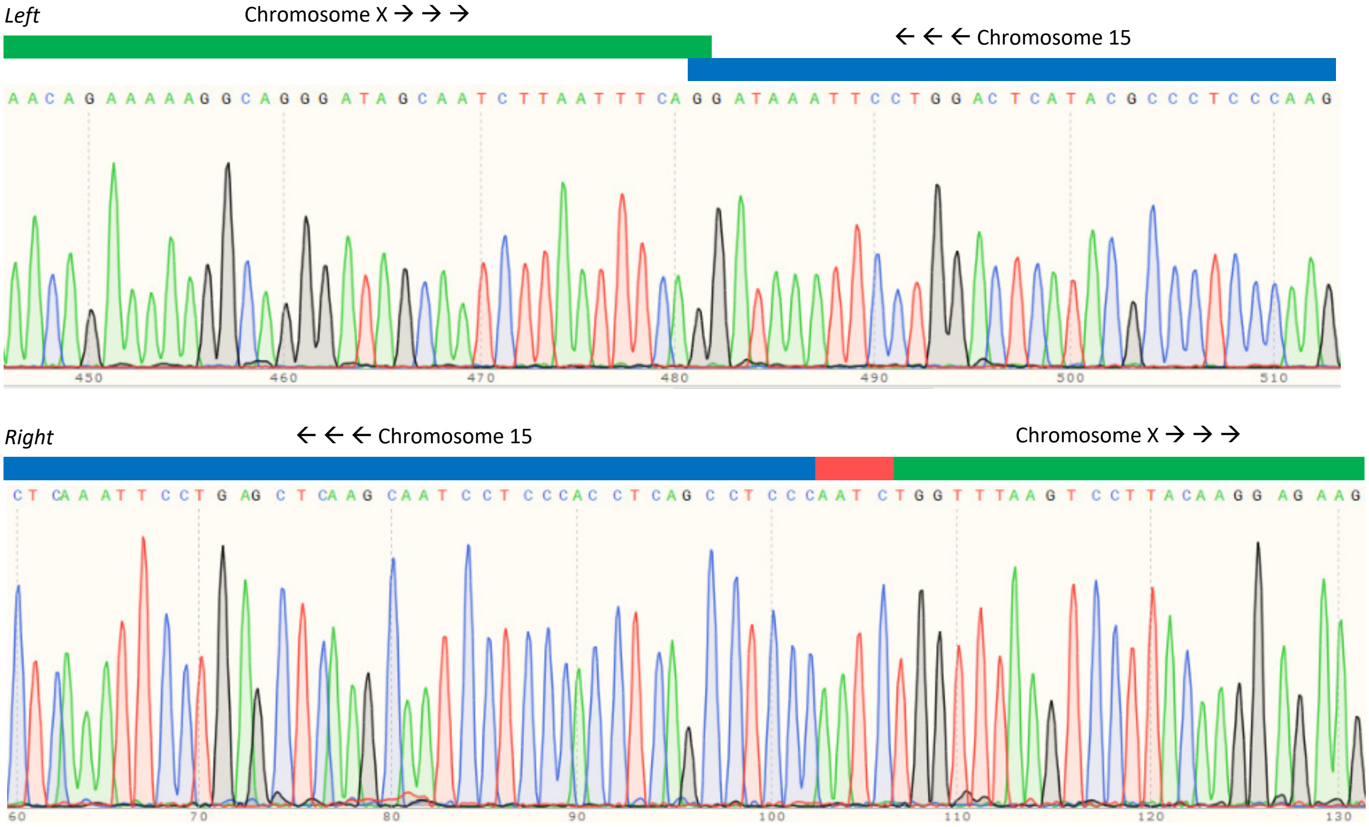

A) PCR and gel electrophoresis of distal breakpoint (upper band), taking along exon 3 of B3GALNT2 as PCR control (lower band). The asterisks indicate the 100 bp DNA ladders. B) Sanger sequencing covering the proximal (upper) and distal (lower) breakpoints.

Figure S6: X-inactivation studies are not conclusive in explaining phenotypic variability between female carriers

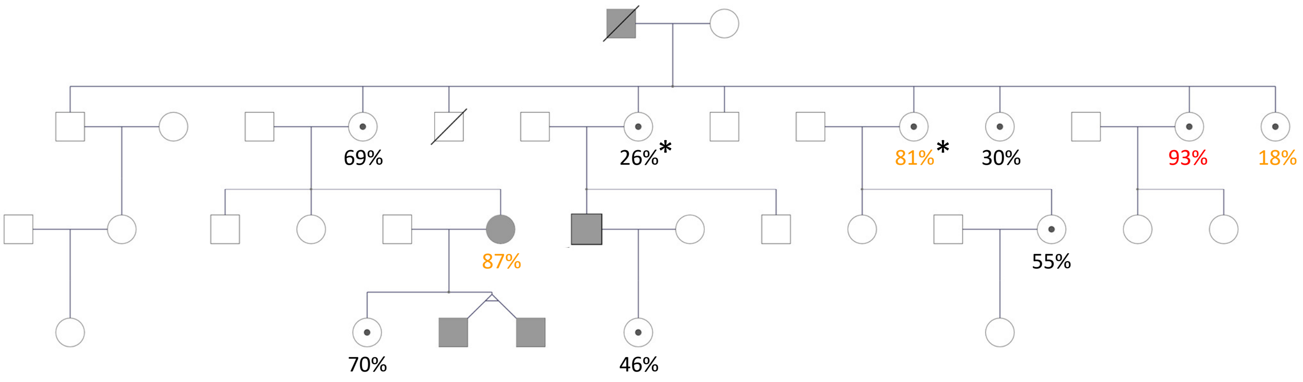

Random X-inactivation	21-79%
Skewed X-inactivation	11-20% and 80-89%
Extremely skewed X-inactivation	≤10% and ≥90%

Pedigree of the family showing the degree of X-inactivation in all female carriers. This analysis indicates random X-inactivation in the majority of female carriers, skewed X-inactivation in three female relatives, including the only mildly affected female (III-6) and two putatively unaffected females, and extreme skewing in one putatively unaffected female carrier. Grey shading indicates individuals are affected, a dot indicates carriership and the asterisk indicates radiographic feet abnormalities.

Figure S7: The SV does not affect *LRRC28* expression

A.

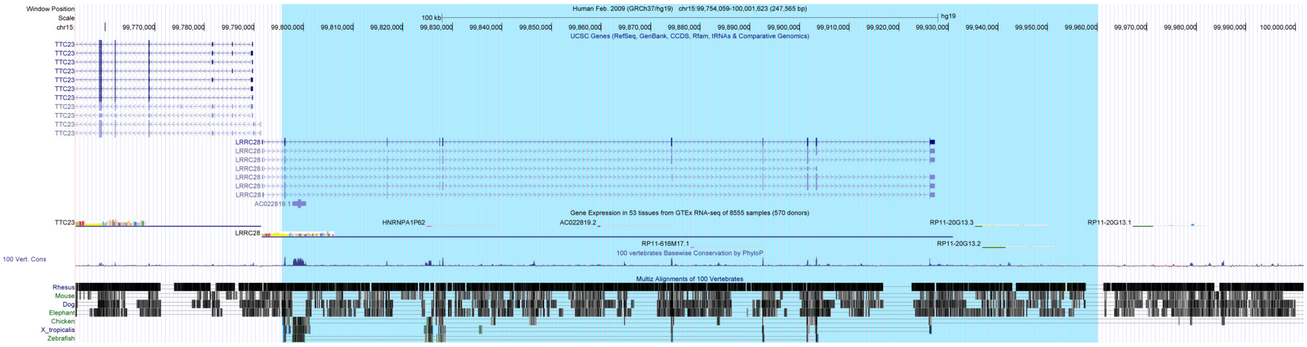

B.

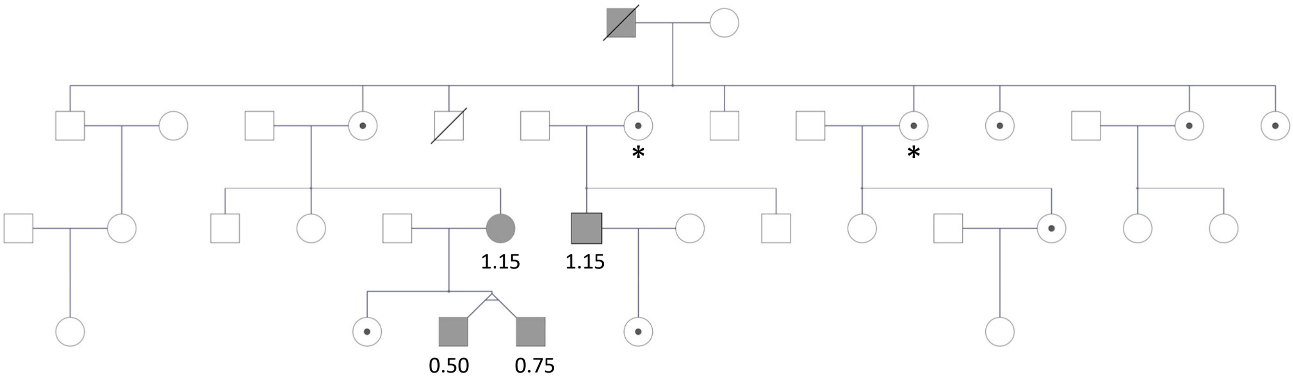

Mean of 6 healthy unrelated controls: 1.02 (range: 0.80-1.38)

C.

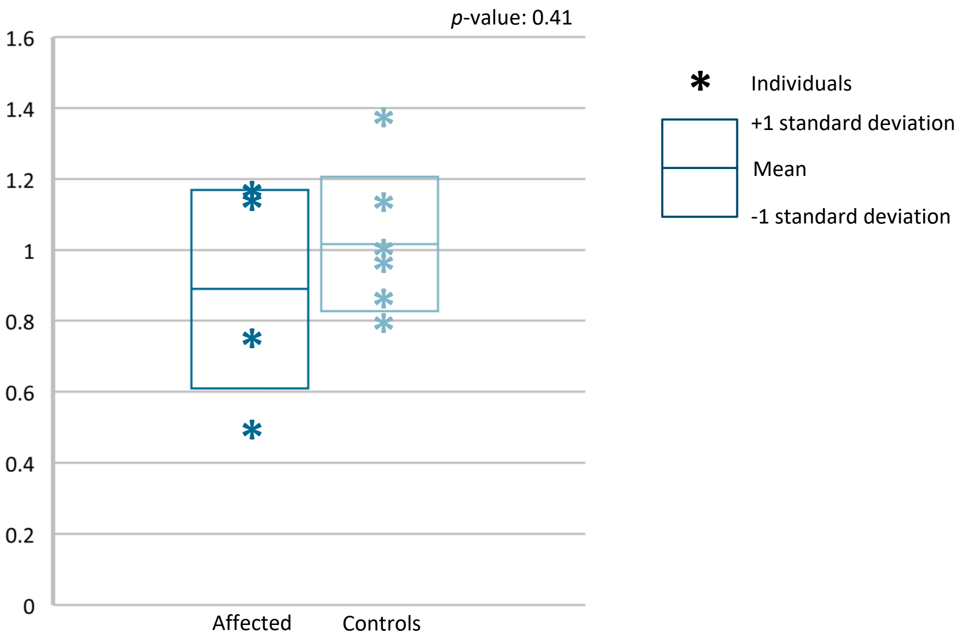

A) Screenshot from UCSC genome browser with gain of 15q26.3 material (blue), showing this gain contains exon 2-10 of LRRC28. B) Relative normalized expression of LRRC28 tested by qPCR on RNA from EBV-LCLs for all individuals for whom EBV-LCLs were available, alongside three healthy male and three female unrelated controls. Grey shading indicates individuals are affected, a dot indicates carriership and the asterisk indicates radiographic feet abnormalities. C) Relative expression of LRRC28 in affected individuals versus controls. The mean and standard deviation are visualized by the boxplot, with measurements per individual indicated by an asterisk.

Figure S8: Genomic 3D organization and TFBS at the locus of the SV and the SHFM2 linkage region on the X-chromosome

A.

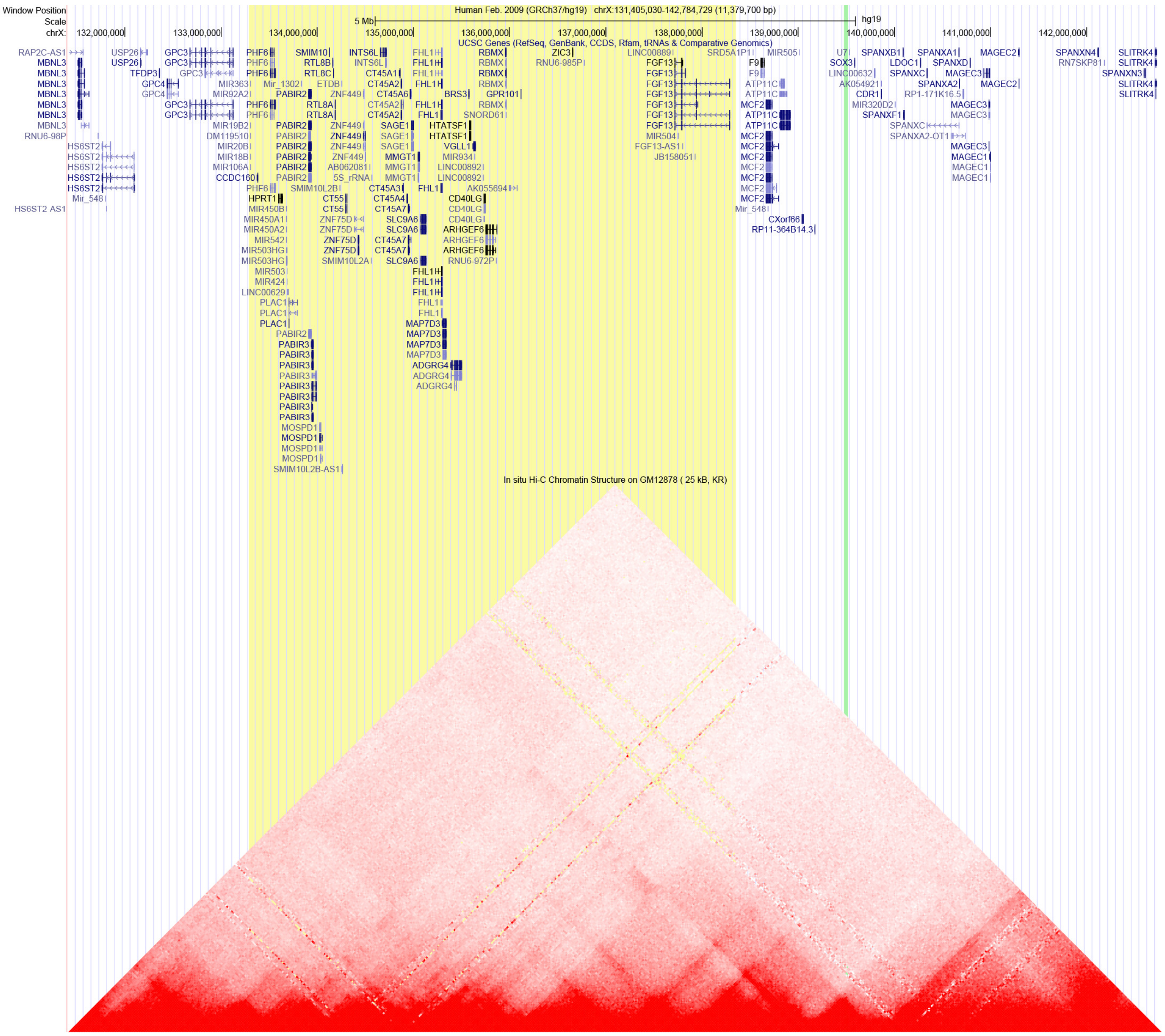

B.

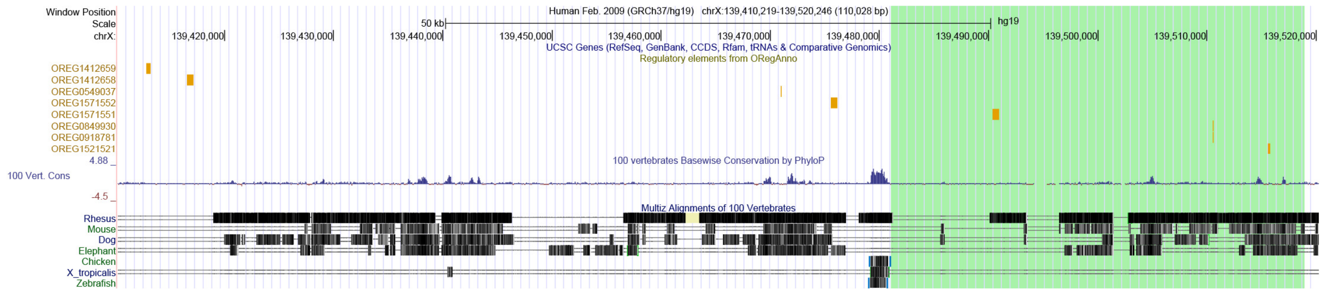

A) Screenshot from the UCSC genome browser with the locus of the SV on the X-chromosome (green) and the SHFM2 linkage region from literature (yellow), showing SOX3 locates downstream of the deletion. The linkage region and the SV are approximately 1.1 Mb apart but partly locate to the same TAD. Based on the distance between the linkage region and the SV, identity by descent is deemed unlikely, although we could not formerly exclude this in the absence of molecular data for the previously published family. However, the fact that both the SV and the linkage region largely locate to the same TAD, suggests the phenotypes observed in the families might be caused by different variants with the same downstream effects. B) Screenshot from the UCSC genome browser showing the deletion of the X-chromosome (green) together with the 100 kb region proximal of the deletion. The deletion and the 100 kb proximal to the SV both contain four TFBS, of which several have SOX3 as target gene. These include OREG1412659 (TFBS of E2F1), OREG1412658 (TFBS of E2F1), OREG1571552 (TFBS of FOXA1), OREG1571551 (TFBS of FOXA1), OREG1521521 (TFBS of ESR1).

Figure S9: An enhancer is included in the duplicated chromosome 15 region

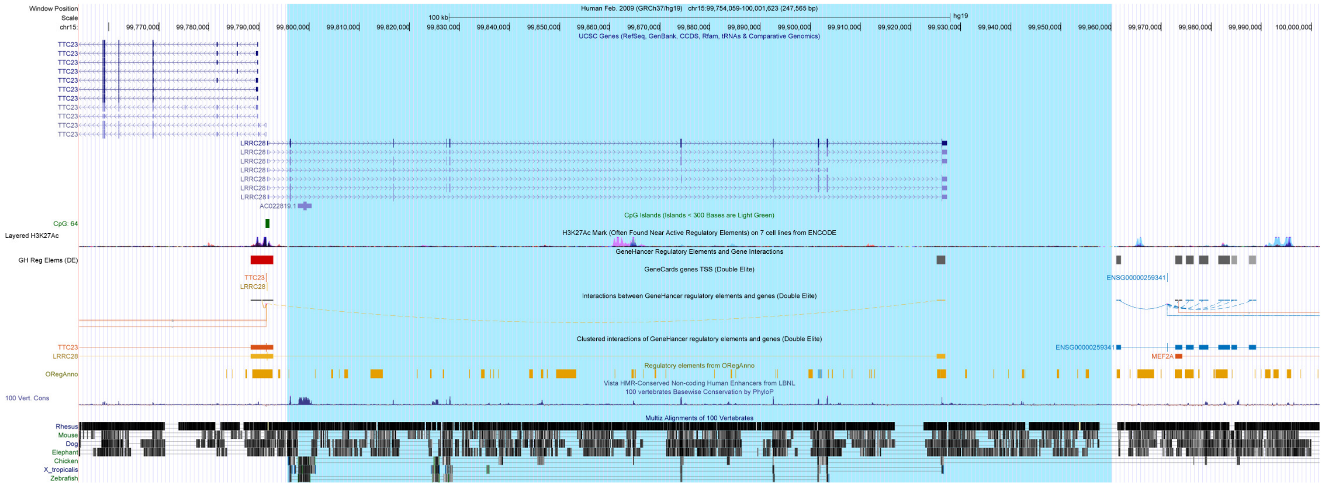

Screenshot from the UCSC genome browser showing the duplicated fragment of chromosome 15 (blue). The gain contains an enhancer that normally interacts with the promoter of LRRC28.

Supplemental information

Materials and methods

Individuals and consent

All affected individuals provided written informed consent to be included in this study. For publication of clinical photographs and radiological imaging, additional consent for photo publication was obtained. All consent procedures are in accordance with both the local ethical guidelines, and the Declaration of Helsinki. Clinical characterization was performed by reviewing the medical files and radiological imaging data and/or by revising the phenotypes of the individuals in the clinic. The study was approved by the Radboudumc local ethics board (2019-5554).

Microarray analysis

Microarray analysis was performed with the Affymetrix CytoScan HD (2.6M) array platform following the manufacturer's specification (Thermo Fisher Scientific, Waltham, USA). Experiments and interpretation of results were performed in the diagnostic workflow with an estimated average resolution of 20 kb on genome build GRCh37/hg19 (1).

Karyotyping and Fluorescence in Situ Hybridization

Karyotyping was performed on cultured EBV-LCLs cells in the diagnostic setting following previously described standard protocols (2), and the International System for Human Cytogenetic Nomenclature (ISCN, 2020) was used to describe chromosomal abnormalities. Chromosome slides were made according to routine procedures. Metaphases were analysed after GTG-banding and FISH experiments were performed on chromosome slides using the probe RP11-668P3 specific for 15q26.3 (Empire Genomics, Williamsville, NY, USA), and probes CEP 15 and CEP X (Vysis, Abbott, Abbott Park, IL, USA) for centromeres of chromosome 15 and chromosome X respectively.

Ultra-high molecular weight DNA extraction

Ultra-high molecular weight (UHMW) DNA was isolated from 1-1.5 million cultured EBV-LCLs, with the SP Blood & Cell Culture DNA Isolation Kit following the manufacturers' instructions (Bionano Genomics®, San Diego, CA, USA) as previously described. In brief, genomic DNA was released by treating cells with LBB lysis buffer, and bound to a nanobind disk, followed by washing steps and elution in the provided elution buffer (3).

Bionano optical genome mapping (Saphyr system)

The DLS (Direct Label and Stain) DNA Labeling Kit (Bionano Genomics®, San Diego, CA, USA) was used to label the UHMW DNA molecules. 750 ng of genomic DNA was labelled with Direct Label Enzyme (DLE-1) and DL-Green fluorophores. DL-Green fluorophores excess was washed out, followed by an overnight DNA backbone counterstaining. The labelled UHMW genomic DNA was loaded on a Saphyr chip® for linearization and visualisation with the Saphyr system (Bionano Genomics, San Diego USA). The Bionano Solve software version v3.6.1 executed the *de novo* assembly and Variant Annotation Pipelines. Output was analysed by a CNV and a SV pipeline, enabling detection of unbalanced aberrations based on differences in normalized molecule coverage, and detection of structural variants based on comparison of labelling patterns between the sample genome map and a reference genome respectively. Interpretation of results was performed using Bionano Access software v1.6.1. To filter on quality of results, confidence values were set as follows: for insertion/deletion=0, inversion=0.01, duplications= -1, translocation=0 and CNV=0. For SV calls, an optical mapping dataset of 204 human control samples (provided by Bionano Genomics) was used to filter out common variants (3).

Whole genome sequencing (WGS)

WGS was outsourced to BGI (BGI, Hongkong, China) on a BGISeq500 sequencing platform using a paired-end module of 2x 100 base pairs with a minimal median coverage of 30-fold per genome.

BWA V.0.78 was used for read mapping to the GRCh37/hg19 reference genome build and bam quality control was performed with Qualimap V.2.2.1. To ensure data quality, several quality metrics were checked, including insert size, percentage mapped reads, percentage duplicated mapped reads, coverage, percentage of bases with more than 20-fold coverage and error rate. The resulting alignment files were subjected to several variant calling pipelines. Variant calling of single nucleotide variants and small indels (SNVs/indels) was carried out using xAtlas V.0.1 and variants were subsequently annotated with an inhouse developed annotation pipeline, that uses the Variant Effect Predictor (VEP V.91) and Gencode V.34lift37 basic gene annotations. Additionally, information on population allele frequency was added from GnomAD V.2.1.1 and from an inhouse database. For genetic variants in genes associated with a known disease, inhouse gene panel information was added. Other included annotations were CADD score V.1.6, SpliceAI, OMIM or KEGG pathways. Detection of Runs of Homozygosity was performed using Plink V.1.07 applying the following parameters: homozyg-window-het=3, homozyg-snp=50 and homozyg-kb=300. Known pathogenic short tandem repeats (STRs) were analysed with Expansion Hunter V.3.1.2. using default settings. CNVs were identified with two CNV calling algorithms, being Control-FREEC (4) and Canvas Copy Number Variant Caller (5), which both use read depth to detect copy number changes. SV calling was performed using the Manta Structural Variant Caller V.1.1.0 (6), that uses a paired-end and split read evidence approach to identify SVs. CNVs and SVs were annotated with an inhouse developed pipeline, based on ANNOVAR (7) and Gencode V.34lift37 basic gene annotations. Additional information on population allele frequency was added from GnomAD V.2.1, 1000G V.8 and GoNL SV database. SNVs/indels, CNVs and SVs were prioritized from WGS by applying a customized inhouse pipeline. Additionally, SNVs/indels were assessed by a phenotype-based variant prioritization using the Exomiser software package (version 13.1.0 with 2202 databases) with a default presets for both exome and genome analyses (8, 9). Prioritized variants were visually inspected in IGV (10).

Validation and segregation with (nested) PCR, Agarose Gel Electrophoresis and Sanger sequencing

The variant was validated by breakpoint-spanning PCRs and evaluation by Agarose Gel Electrophoresis in all individuals with DNA available. For amplification of the left breakpoint, long-range PCR (LR-PCR) followed by a nested conventional PCR was performed. For the right breakpoint, a conventional PCR was applied for amplification, also including a control PCR reaction for *B3GALNT2.* Primers were designed using Primer3web v4.1.0 software. LR-PCR and (nested) conventional PCRs were performed according to the manufacturer's protocols using the Q5 High-Fidelity 2X Master Mix (New England BioLabs inc.) and the Amplitaq Gold 360 Master Mix (Thermo Fisher Scientific) respectively. For segregation analysis, the agarose gels of both breakpoints were inspected for the presence or absence of a PCR product of the expected size. For individual III-7, the PCR products were enzymatically cleaned with Exonuclease I and FastAP, followed by Sanger sequencing using a routine diagnostic workflow (11). Finally, Sanger sequencing traces were analysed using the Chromas Lite v2.1.1 software package (Technelysium) to verify the exact sequence at both breakpoints that was seen in WGS data.

qPCR for LRRC28, SOX3 and FGF13 expression

For the quantitative real-time PCR (qPCR) experiments, RNA was isolated from cultured EBV-LCLs from four affected individuals and six controls following the NuleoSpin RNA isolation protocol (Machery-Nagel). RNA was converted to cDNA with the iScript cDNA Synthesis Kit (BioRad). qPCR primers were designed with Primer3 v4.1.0 software. qPCR was performed according to standard protocol with GoTaq 2x master mix (Promega). *GUSB* was included in the qPCR experiments as reference gene. For all primers, standard curves were made with a cDNA control sample in a dilution series (20x, 80x, 320x, 1280x, 5120x dilutions in MilliQ). For all qPCR experiments a blank sample was taken along for each primer pair, and all samples were tested in duplicate. Relative expression of the genes of interest (*LRRC28, SOX3* and *FGF13)* was calculated by normalizing Ct values for these genes with Ct values of *GUSB* and normalizing individual delta Ct scores to the mean of delta Ct scores of

controls. Relative expression of the genes of interest in affected individuals was compared to controls with a paired T-test. P-values below 0.05 were considered statistically significant.

Primers for PCR and qPCR

**Goal of primer pair**	**Forward primer**	**Reverse primer**	**Product size (bp)**	**Notes**
LR-PCR spanning left breakpoint	GATCGTCTGTGATGGTTAGGTG	GTGATGTCAGCAAGTGGGATAC	7,824	Forward primer specific to the locus on chrX; reverse primer specific to the locus on chr15
Conventional (nested) PCR spanning left breakpoint	GCTAGTAAGGGCACATAGAGC	TGCCTCAATGTTCTTCAGGG	836	Forward primer specific to the locus on chrX; reverse primer aligning to the locus on chr15, but supplementary alignment to different loci
Conventional PCR spanning right breakpoint	TACTATAGAGAGCACCACCACAC	CAGGAGCCACGCACATAATG	426	Forward primer specific to the locus on chr15; reverse primer specific to the locus on chrX
PCR control using exon 3 of *B3GALNT2*	AAATGGGCATGAGGAAACG	AAGCTTAGCAACTTTTACTCAACATC	238	
qPCR on exon 5 of *LRRC28* (gene of interest)	CCATTGGGTCTCTTGTAAAACTC	TCGAAGATGACGTAAAGCTCTC	104	
qPCR on *SOX3* (gene of interest)	TGGAGAACTGCAACGCCTACGC	GATCACGGCAGAAATCACCAACTC	204	Primers as in (12)
qPCR on *FGF13* (gene of interest)	CAGCCGACAAGGCTACCAC	GTTCCGAGGTGTACAAGTATCC	185	Primers as in (12)
qPCR on *GUSB* (gene for normalization)	CTGTACACGACACCCACCAC	TACAGATAGGCAGGGCGTTC	245	

X-inactivation studies

X-inactivation studies were performed in the diagnostic workflow using DNA derived from blood of all female SV carriers as previously described (13).

In silico analysis of regulatory functions

Regulatory functions of the regions affected by the SV were assessed using the UCSC genome browser (14), visualizing datasets from the GeneHancer database (15) and the Open Regulatory Annotation database (ORegAnno; http://www.oreganno.org/dump/) (16). To assess TFBS in the SV,

we downloaded the complete dataset from ORegAnno (ORegAnno_Combined_2014.09.15.tsv), that also includes data from PAZAR and JASPAR databases (16-18). Additionally, candidate cis-Regulatory Elements that are active in the human developing limb were examined using SCREEN: Search Candidate cis-Regulatory Elements by ENCODE (Registry of cCREs V3) (19) after liftover of the relevant sequences to GRCh38/hg38 (chrX:140398896-140436824 and chr15:99255115-99420157).

Supplemental results

X-inactivation studies

We performed X-inactivation studies by quantifying methylation of the human androgen receptor locus including all ten confirmed female carriers (II-4, II-7, II-10, II-11, II-13, II-14, III-6, III-12, IV-2 and IV-5). We found that the majority of these individuals exhibited random X-inactivation (21-79%; individual II-4, II-7, II-11, III-12, IV-2 and IV-5), three individuals showed skewed X-inactivation (11-20% and 80-89%; individual II-10, II-14 and III-6) and only in individual II-13 extreme skewing of X-inactivation was seen (≤10% and ≥90%; Figure S6). Whereas these results do not explain why III-6 is the only female expressing split-hand, we can conclude that increased X-chromosome inactivation is not the protective mechanism for non-penetrance, as is illustrated by the female carriers with random X-inactivation without any limb abnormalities. Although our experimental set-up did not prove which X-chromosome was inactivated (e.g. the one with or without the SV) and interpretation of X-inactivation studies has intrinsic challenges (20-23), our results could partially be explained if individual III-6 would have skewed inactivation of the X-chromosome without the SV. This would make the X-chromosome containing the SV more active, resulting in individual III-6 having a mild phenotype, whereas in theory the other female carriers might show (extreme) skewing of the X-chromosome containing the SV. This would explain why they do not exhibit any limb abnormalities, although the subclinical foot abnormalities and random X-inactivation in individual II-7 do not fit with this hypothesis. Alternatively, it is not unthinkable that – like individual II-10 – individuals II-13 and II-

14 are subclinically or mildly affected and/or that the available information obtained per family history is incomplete, which would also largely explain our observations.

Web resources

https://omim.org/

http://www.oreganno.org/dump/

https://screen.wenglab.org/

Supplemental references

1. de Ligt J, Boone PM, Pfundt R, Vissers LE, de Leeuw N, Shaw C, et al. Platform comparison of detecting copy number variants with microarrays and whole-exome sequencing. Genom Data. 2014;2:144-6.
2. Bates SE. Classical cytogenetics: karyotyping techniques. Methods Mol Biol. 2011;767:177-90.
3. Mantere T, Neveling K, Pebrel-Richard C, Benoist M, van der Zande G, Kater-Baats E, et al. Optical genome mapping enables constitutional chromosomal aberration detection. Am J Hum Genet. 2021.
4. Boeva V, Popova T, Bleakley K, Chiche P, Cappo J, Schleiermacher G, et al. Control-FREEC: a tool for assessing copy number and allelic content using next-generation sequencing data. Bioinformatics. 2012;28(3):423-5.
5. Roller E, Ivakhno S, Lee S, Royce T, Tanner S. Canvas: versatile and scalable detection of copy number variants. Bioinformatics. 2016;32(15):2375-7.
6. Chen X, Schulz-Trieglaff O, Shaw R, Barnes B, Schlesinger F, Källberg M, et al. Manta: rapid detection of structural variants and indels for germline and cancer sequencing applications. Bioinformatics. 2016;32(8):1220-2.
7. Wang K, Li M, Hakonarson H. ANNOVAR: functional annotation of genetic variants from high-throughput sequencing data. Nucleic Acids Res. 2010;38(16):e164.
8. Jacobsen JOB, Kelly C, Cipriani V, Robinson PN, Smedley D. Evaluation of phenotype-driven gene prioritization methods for Mendelian diseases. Brief Bioinform. 2022;23(5).
9. Smedley D, Jacobsen JO, Jäger M, Köhler S, Holtgrewe M, Schubach M, et al. Next-generation diagnostics and disease-gene discovery with the Exomiser. Nat Protoc. 2015;10(12):2004-15.
10. Robinson JT, Thorvaldsdóttir H, Winckler W, Guttman M, Lander ES, Getz G, et al. Integrative genomics viewer. Nat Biotechnol. 2011;29(1):24-6.
11. de Ligt J, Willemsen MH, van Bon BW, Kleefstra T, Yntema HG, Kroes T, et al. Diagnostic exome sequencing in persons with severe intellectual disability. N Engl J Med. 2012;367(20):1921-9.
12. Haines B, Hughes J, Corbett M, Shaw M, Innes J, Patel L, et al. Interchromosomal insertional translocation at Xq26.3 alters SOX3 expression in an individual with XX male sex reversal. J Clin Endocrinol Metab. 2015;100(5):E815-20.

13. Allen RC, Zoghbi HY, Moseley AB, Rosenblatt HM, Belmont JW. Methylation of HpaII and HhaI sites near the polymorphic CAG repeat in the human androgen-receptor gene correlates with X chromosome inactivation. Am J Hum Genet. 1992;51(6):1229-39.
14. Lee BT, Barber GP, Benet-Pagès A, Casper J, Clawson H, Diekhans M, et al. The UCSC Genome Browser database: 2022 update. Nucleic Acids Res. 2022;50(D1):D1115-d22.
15. Fishilevich S, Nudel R, Rappaport N, Hadar R, Plaschkes I, Iny Stein T, et al. GeneHancer: genome-wide integration of enhancers and target genes in GeneCards. Database (Oxford). 2017;2017.
16. Lesurf R, Cotto KC, Wang G, Griffith M, Kasaian K, Jones SJ, et al. ORegAnno 3.0: a community-driven resource for curated regulatory annotation. Nucleic Acids Res. 2016;44(D1):D126-32.
17. Portales-Casamar E, Arenillas D, Lim J, Swanson MI, Jiang S, McCallum A, et al. The PAZAR database of gene regulatory information coupled to the ORCA toolkit for the study of regulatory sequences. Nucleic Acids Res. 2009;37(Database issue):D54-60.
18. Mathelier A, Zhao X, Zhang AW, Parcy F, Worsley-Hunt R, Arenillas DJ, et al. JASPAR 2014: an extensively expanded and updated open-access database of transcription factor binding profiles. Nucleic Acids Res. 2014;42(Database issue):D142-7.
19. Moore JE, Purcaro MJ, Pratt HE, Epstein CB, Shoresh N, Adrian J, et al. Expanded encyclopaedias of DNA elements in the human and mouse genomes. Nature. 2020;583(7818):699-710.
20. Orstavik KH. X chromosome inactivation in clinical practice. Hum Genet. 2009;126(3):363-73.
21. Sharp A, Robinson D, Jacobs P. Age- and tissue-specific variation of X chromosome inactivation ratios in normal women. Hum Genet. 2000;107(4):343-9.
22. Shvetsova E, Sofronova A, Monajemi R, Gagalova K, Draisma HHM, White SJ, et al. Skewed X-inactivation is common in the general female population. Eur J Hum Genet. 2019;27(3):455-65.
23. Amos-Landgraf JM, Cottle A, Plenge RM, Friez M, Schwartz CE, Longshore J, et al. X chromosome-inactivation patterns of 1,005 phenotypically unaffected females. Am J Hum Genet. 2006;79(3):493-9.
